# Efficacy and safety of vitamin C supplementation in the treatment of community-acquired pneumonia: a systematic review and meta-analysis with trial sequential analysis

**DOI:** 10.1038/s41598-024-62571-5

**Published:** 2024-05-24

**Authors:** Yogesh Sharma, Subodha Sumanadasa, Rashmi Shahi, Richard Woodman, Arduino A. Mangoni, Shailesh Bihari, Campbell Thompson

**Affiliations:** 1 College of Medicine and Public Health, Flinders University, Adelaide, SA 5042 Australia; 2 Division of Medicine, Cardiac & Critical Care, Flinders Medical Centre, Adelaide, SA 5042 Australia; 3 Flinders Medical Centre, Adelaide, SA 5042 Australia; 4 College of Medicine and Public Health, Flinders University, Adelaide, SA 5042 Australia; 5 Department of Biostatistics, College of Medicine and Public Health, Flinders University, Adelaide, SA 5042 Australia; 6 Discipline of Clinical Pharmacology, College of Medicine and Public Health, Flinders University, Adelaide, SA 5042 Australia; 7 College of Medicine and Public Health, Flinders University, Adelaide, SA 5042 Australia; 8 Discipline of Medicine, The University of Adelaide, Adelaide, SA 5005 Australia

**Keywords:** Respiratory tract diseases, Bacteria, Virology

## Abstract

Community-acquired pneumonia (CAP) poses a significant global health challenge, prompting exploration of innovative treatments. This systematic review and meta-analysis aimed to evaluate the efficacy and safety of vitamin C supplementation in adults undergoing treatment for CAP. A comprehensive search of the MEDLINE, Embase, CINAHL, the Cochrane Central Register of Controlled Trials, and Clinical Trials.gov databases from inception to 17 November 2023 identified six randomized-controlled-trials (RCTs) meeting inclusion criteria. The primary outcome analysis revealed a non-significant trend towards reduced overall mortality in the vitamin C group compared to controls (RR 0.51; 95% CI 0.24 to 1.09; p = 0.052; *I*^2^ = *0*; p = 0.65). Sensitivity analysis, excluding corona-virus-disease 2019 (COVID-19) studies and considering the route of vitamin C administration, confirmed this trend. Secondary outcomes, including hospital length-of-stay (LOS), intensive-care-unit (ICU) LOS, and mechanical ventilation, exhibited mixed results. Notably, heterogeneity and publication bias were observed in hospital LOS analysis, necessitating cautious interpretation. Adverse effects were minimal, with isolated incidents of nausea, vomiting, hypotension, and tachycardia reported. This meta-analysis suggests potential benefits of vitamin C supplementation in CAP treatment. However, inconclusive findings and methodological limitations warrants cautious interpretation, emphasising the urgency for high-quality trials to elucidate the true impact of vitamin C supplementation in CAP management.

## Introduction

Community acquired pneumonia (CAP) is defined as an acute infection of the pulmonary parenchyma acquired outside hospital and is a leading cause of morbidity and mortality worldwide. Globally, CAP is the second most common cause of hospitalisation and is the most common infectious cause of death^1^. According to the World Health Organisation (WHO)^2^, lower respiratory tract infections remain the primary infective cause of death globally accounting for 6.1% of deaths.

Inpatient mortality from CAP ranges between 4.2 and 5.5% while mortality at 6 months can be as high as 23%^3^. Recent evidence^4^ suggest that despite advancements in clinical care, mortality rates from pneumonia have not any shown any substantial change over time. An excess inflammatory response seems to be partly responsible for treatment failure in some patients with CAP and has been associated with poor clinical response to antibiotics^5^. Therefore, there is a need to explore adjunctive therapies that have immunomodulatory and barrier-enhancing functions augmenting treatment of CAP.

Vitamin C is a water-soluble vitamin with powerful antioxidant properties that can scavenge free radicals^6^. This vitamin has immune mediating properties as it has been found to support neutrophil migration to the site of infection and is responsible for production of hormones such as noradrenaline and vasopressin^6^. These properties have led to an investigation of its potential role as an additional therapeutic agent in the treatment of pneumonia.

Clinical studies of vitamin C supplementation in pneumonia have yielded varied results. While some studies^7,8^ have suggested that vitamin C supplementation may reduce severity of pneumonia^7^, the impact on mortality remains unclear with one study^9^ suggesting a significant reduction in mortality while the other^10^ showing no difference in mortality but a trend towards reduction of hospital length of stay (LOS). In addition, the safety of vitamin C in CAP remains unclear. Therefore, we conducted a systematic review to assess the efficacy and safety of parenteral and or oral vitamin C alone or in combination with other therapies in adults being treated for CAP.

## Materials and methods

This systematic review and meta-analysis adheres to the Preferred Reporting Items for Systematic Reviews and Meta-Analysis Protocols (PRISMA) 2020 standards. The research protocol was registered with the International Prospective Registry of Systematic Reviews (PROSPERO) number CRD42023483860.

### Search strategy

We searched the following electronic databases: MEDLINE, Embase, CINAHL, the Cochrane Central Register of Controlled Trials, and Clinical Trials.gov from inception to 17 November 2023 with the help of a medical librarian. The search strategy for this systematic review is provided in Supplementary File 2. We used a combination of keywords and medical subject headings (MeSH) as follows: adults, community acquired pneumonia, bronchopneumonia, lower respiratory tract infections, hospitalisation, inpatients, critical care, vitamin C, ascorbic acid, ascorbate, mortality, randomised controlled trials (RCTs), placebo, intravenous administration and oral vitamin C. No language restrictions were applied.

### Eligibility criteria

#### Design and population

We included parallel-arm RCTs of adults aged ≥ 18 years with CAP. Pneumonia was defined as symptoms of fever, dyspnoea, cough, and sputum production along with imaging evidence of a pulmonary infiltrate requiring hospitalisation and possibly intensive care unit (ICU) admission. We included publications in which authors did not clearly define pneumonia but instead used terms such as 'pneumonia' or ‘consolidation on imaging studies' to identify their target population.

### Intervention

Clinical trials with at least one arm involving the administration of parenteral and or oral vitamin C alone or in combination with other micronutrients and therapies were included.

### Comparator arm

We included studies which had at least one control arm which included patients who were not prescribed parenteral and or oral vitamin C. The control arm may have received placebo or any other active treatment.

### Types of outcome measures

#### Data extraction

Two reviewers (YS and SS) screened identified citations at the title and abstract screening level using predefined eligibility criteria electronically by use of reference manager. Potentially eligible citations were then reviewed at the level of full-text screening by the paired reviewers. The screening was completely independent and in duplicate and any disagreements were resolved by involvement of a third reviewer (RS). We included studies based upon the eligibility criteria and reporting at least one primary or secondary outcome of interest.

### Study quality assessment

The quality of studies was independently assessed by two reviewers (YS and RS) who evaluated the risk of bias using the modified version of the RoB tool (Rob 2.0)^11^ and the modified Jadad scale^12^. Risk of bias were classified as low risk, high risk or unclear risk after assessment of the following key domains: generation of random sequence, use of allocation concealment method, blinding of participants, data collectors, and outcome assessors, and incomplete or missing outcome data and other biases. In addition, the quality of studies was independently assessed by the two reviewers by using the modified Jadad scale. Studies with a modified Jadad scale score of 1–3 are considered low-quality studies and those with a score of 4- 7 were considered as high quality studies^12^.

### Outcome measures

#### Primary outcomes

The primary outcome was overall mortality from date of admission including in-hospital deaths. Different studies have used in-hospital mortality or 30-day mortality. We included mortality data closest to the time points of interest.

#### Secondary outcomes

The secondary outcomes included length of hospital stay (LOS), intensive care unit (ICU) LOS, 30-day readmission risk, use of vasopressor support, use of non-invasive and invasive ventilation, time to clinical stabilisation (defined previously^13^ as patients achieving all the following criteria: (1) temperature ≤ 37.8 °C; (2) heart rate ≤ 100 beats/minute; (3) respiratory rate ≤ 24 breaths/minute; (4) systolic blood pressure ≥ 90 mmHg; and (5) arterial oxygen saturation ≥ 90% or partial pressure of oxygen ≥ 60 mmHg on room air), and adverse events relating to the use of vitamin C.

### Effect measures

Binary outcomes were reported as relative risks (RR), while continuous outcomes as standardised mean differences (SMD) with their corresponding 95% confidence intervals (CI).

### Statistical analyses

For data processing we converted medians and interquartile ranges (IQR) to means and standard deviations (SD) as suggested by the Cochrane Collaboration Group^14^. The interventions were compared by use of the random effects modelling and Forest plots were generated. The statistical heterogeneity among studies was assessed by use of the chi-squared test and the *I*^2^ statistics. If significant heterogeneity was detected, then a leave-one-out sensitivity analysis^15^ was performed in STATA to evaluate the influence of individual studies on the pooled estimate. Publication bias was assessed by visual inspection of the funnel plots and use of the Egger’s test for small-study effects^16^. In case of fewer than 10 studies, Egger’s test reliability is compromised. In such instances, a fail-safe calculation following the Rosenthal approach^17^ estimated additional studies needed to assess and mitigate potential publication bias. All statistical analyses were performed by use of Stata software version 18.0 and all estimates were reported with a 95% CI.

### Sensitivity analyses

We performed sensitivity analysis after excluding: (1) studies which included only COVID-19 positive patients (as diagnosed by a positive viral reverse transcription polymerase chain reaction (RT-PCR) test results), and (2) studies which used only oral preparations of vitamin C, to determine the differential impact of vitamin C on mortality among CAP patients according to their COVID status and route of vitamin C administration, respectively. In addition, if significant heterogeneity was observed in the included studies, then further exploration was done by use of a leave-one-out sensitivity analysis using STATA.

### Trial sequential analysis (TSA)

We conducted a TSA for overall mortality to control for both type-1 and type-2 errors and to further validate the findings of our meta-analysis^18^. The chosen parameters for this analysis were alpha = 5% and beta = 20%. The DerSimonian–Laird random effects model was employed, with between-trial heterogeneity adjusted by the diversity-estimate (*D*^2^)^19^. We used the control group mortality of 15.2%, as determined by this meta-analysis, and the effect size (relative risk reduction (RRR)) of 40% as observed in a previous meta-analysis^20^. Sensitivity analyses were also performed for RRRs of 30% and 20%, respectively.

Additionally, a sensitivity analysis using the Biggerstaff-Tweedie random effects model^21^ was conducted, attributing more weight to larger trials than smaller trials. The TSA data analysis was carried out using TSA software (0.9.5.10 Beta, The Copenhagen Trial Unit, Denmark).

## Results

### Study identification and selection

**Figure 1:**
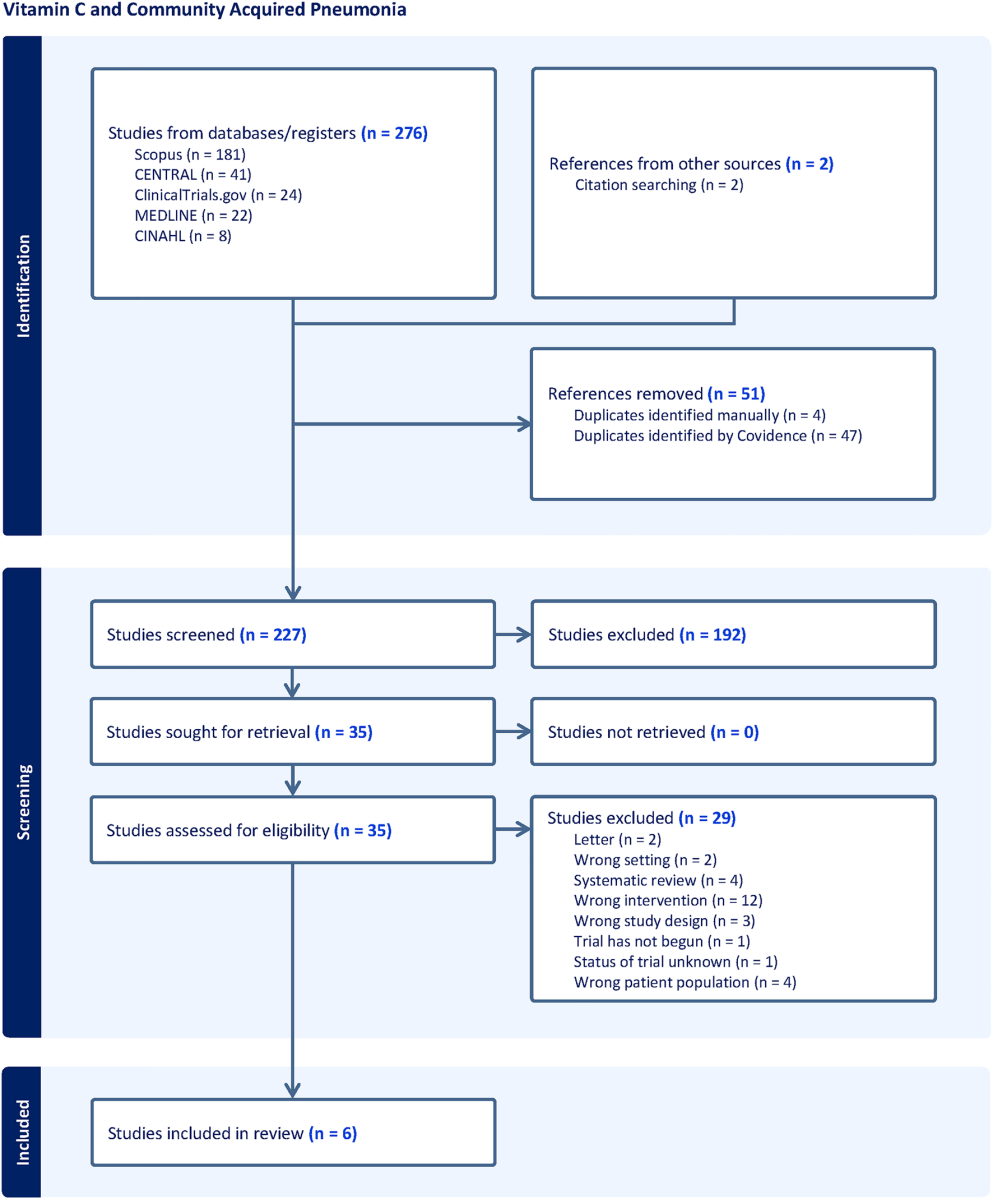
PRISMA flow diagram showing four phases of the study.

**Figure 2:**
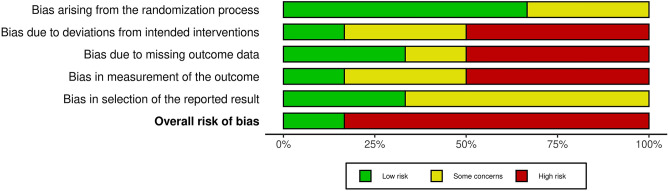
Risk of bias assessment.

Our initial search identified 276 studies from Scopus, Cochrane CENTRAL, ClinicalTrials.gov, MEDLINE and CINHAL (Fig. 1) and 2 studies were identified by manual citation searching. Finally, six eligible studies enrolling a total of 366 patients were included in the meta-analysis^7,10,22–25^. It is noteworthy that two additional studies^26,27^ discovered through manual searches of references were excluded from our review. For detailed information, please refer to Supplementary File 3.

#### Study characteristics

The characteristics of six studies are shown in Table 1. Two studies included only COVID-19 patients^23,25^ while two studies used only oral preparations of vitamin C^7,24^. Four studies^7,10,22,24^ compared vitamin C with a matching placebo and one study^22^ included CAP patients who were admitted in the ICU. Five studies^22–25^ were published in 2021 or later.

**Table 1:** Characteristics of the included studies.

Study and year	Country	Study design	Patients (treatment/control)	Modified Jadad score	Inclusion criteria	Exclusion criteria	Treatment intervention	Control intervention
Hunt et al. 1994^7^	UK	RCT	57 (28/29)	3	Elderly hospitalised patients with acute bronchitis and bronchopneumonia	(1) Suspected or known lung cancer (2) Patients judged at high risk of death within 1–2 days by the clinician	Oral vitamin C tablet twice daily at 8.30am and 2.00 pm for 4 weeks	Matching placebo tablets twice daily at 8.30am and 2.00 pm for 4 weeks
Jamali Moghadam Siahkali et al. 2021^23^	Iran	RCT	60 (30/30)	2	(1) Age > 18 years (2) COVID-19 PCR positive or COVID-19 suspicion based on clinical findings (fever, dysponea and dry cough) (3) Imaging findings of COVID-19 (4) Clinical manifestations of ARDS or myocarditis (5) SaO2 < 93% from admission or after 48 h from first COVID-19 treatment	(1) Received anti-retroviral therapy or immune system booster medications in the last 3-months (2) No proven and confirmed COVID-19 disease (3) G6PD deficiency (4) End-stage renal disease (5) Pregnancy	Vitamin C intravenous 1.5 g 6 hourly for 5 days	Did not receive vitamin C
Mahmoodpoor et al. 2021^22^	Iran	RCT	80 (40/40)	4	ICU patients with severe pneumonia defined as CURB65 score > 3, one major criteria or ≥ 3 minor criteria	(1) less than 18 or more than 80 years (2) Renal insufficiency (3) history of vitamin C use in past 48 h (4) Allergy to vitamin C (5) pregnancy or breast feeding (6) Life expectancy < 24 h (7) End-stage lung disease (8) End-stage malignancy (9) G6PD deficiency (10) Diabetic ketoacidosis (11) Active kidney stone disease	Intravenous vitamin C infusion 60 mg/kg/day for 96 h	Intravenous infusion of normal saline same volume for 96 h
Nikzad et al. 2021^24^	Iran	RCT	40 (20/20)	1	CAP hospitalised patients	(1) Kidney failure (2) Renal failure (3) Taking chemotherapy and anti-inflammatory drugs (4) Antibiotics due to other infections	Oral vitamin C tablet 1000 mg/day for 10 days	Placebo tablets for 10 days
Tehrani et al. 2022^25^	Iran	RCT	54 (18/26)	2	Age ≥ 18 years, RR > 30/min, SaO_2_ < 93%, pulmonary infiltrates, SARS-COV-2 PCR positive	(1) Allergy to vitamin C (2) Cardiogenic pulmonary oedema (3) Pregnancy or breast feeding (4) Chronic renal failure (5) Diabetic ketoacidosis (6) History of nephrolithiasis	Intravenous vitamin C 2 gm every 6 h for 5 days in addition to standard treatment	Standard treatment Hydroxychloroquine 400 mg stat, Interferon beta-1a 44 mcg three times and Lopinavir/Ritonavir 400/100 mg 12 hourly
Chambers et al. 2023^10^	New Zealand	RCT, double blind	75 (36/39)	6	Adults CAP patients ≥ 18 years	1) Admission to hospital > 48 h prior to screening (2) Unable to give informed consent (3) CURB65 pneumonia severity score < 2 (4) Pneumonia not the principal reason for admission (5) Pneumonia associated with bronchial obstruction (6) Bronchiectasis or known tuberculosis (7) Hospital admission in previous two weeks (8) Severe immunosuppression (9) History of nephrolithiasis (10) Renal impairment (eGFR < 30 ml/sec) (11) G6PD deficiency (12) Haemochromatosis (13) Pregnancy or breast feeding	Vitamin C 2.5 g in 100 ml normal saline intravenously over 20–30 min every 8 hourly till attending team changed intravenous antimicrobials to oral therapy. Then oral vitamin C 1 gm three times daily for 7 days	Matching placebo infusion every 8 hourly till attending team changed intravenous to oral antimicrobials to oral therapy. Then oral placebo tablets three times daily for 7 days

### Study quality assessment

Assessment of the quality of the included studies based on the Cochrane Collaboration’s Tool is shown in Fig. 2 and the scores of the modified Jadad scale are presented in Table 1. Apart from two studies^10,22^, all studies were graded low quality according to the assessment tools.

### Primary outcome

#### Overall mortality

**Figure 3:**
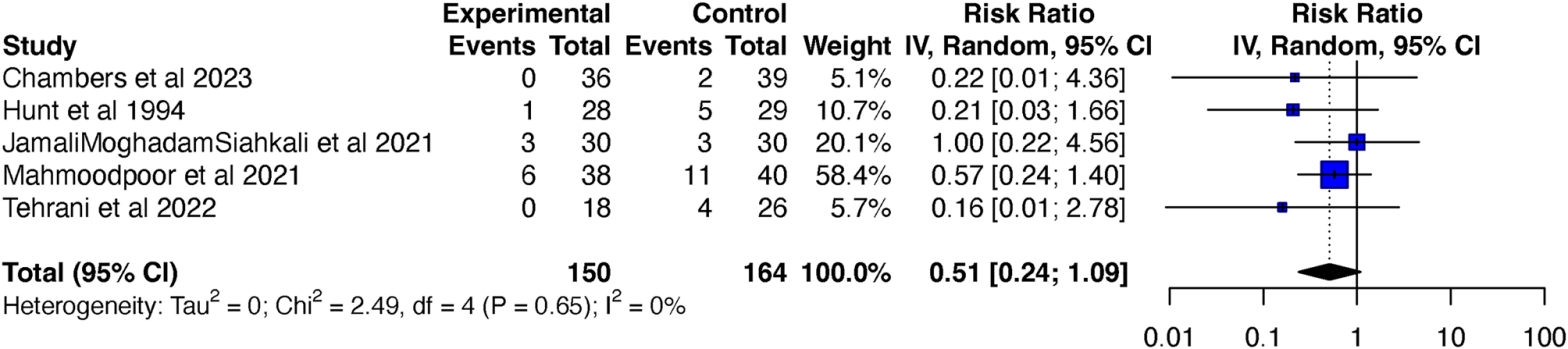
Forest plot showing comparison of overall mortality between vitamin C supplemented group and control group. *CI* confidence interval.

**Figure 4:**
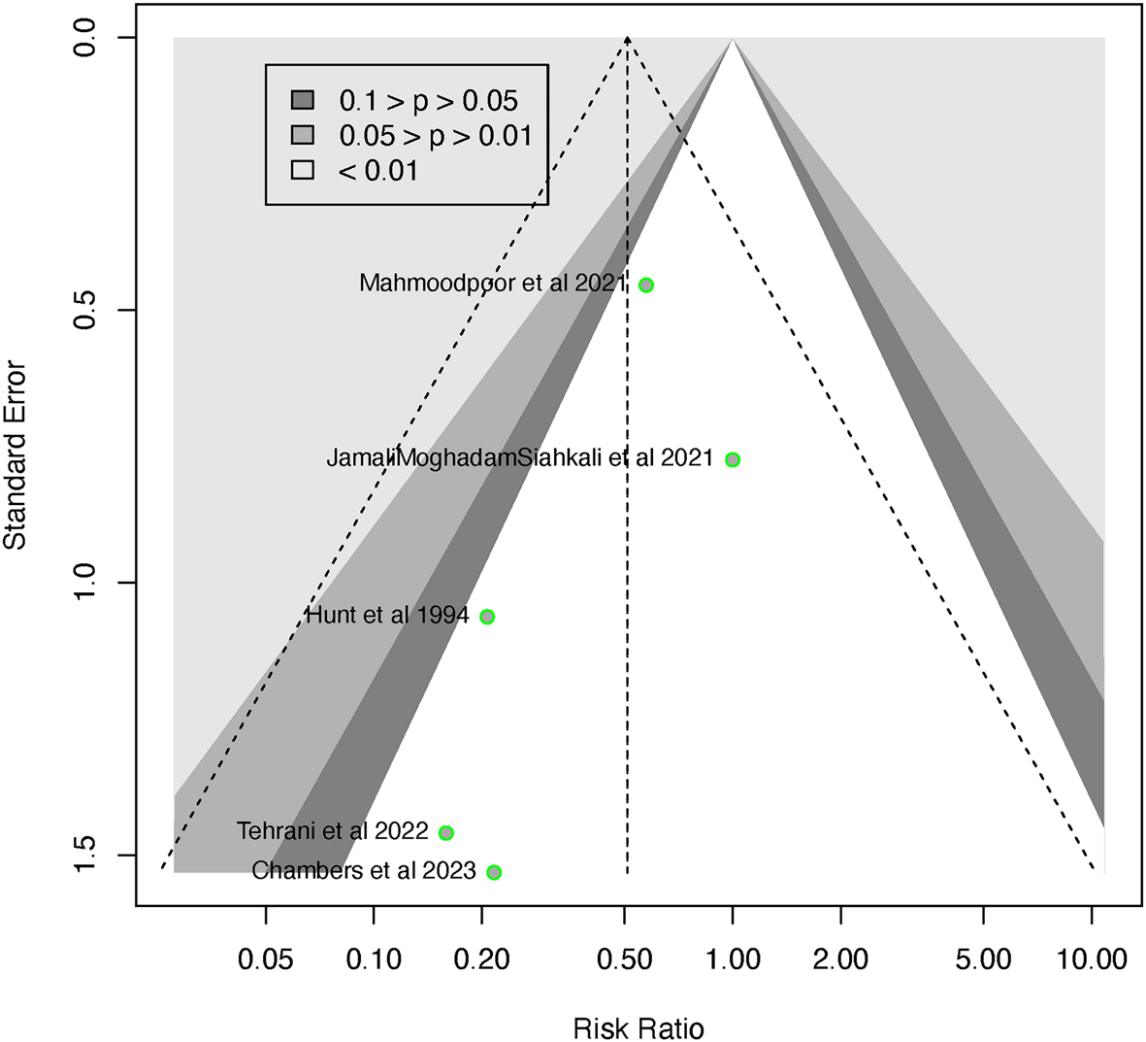
Funnel plot for overall mortality.

Five of the six studies^7,10,22,23,25^ were included in the analysis for overall mortality, consisting of 314 patients, which included 150 patients in the vitamin C supplemented group and 164 patients in the control group. The overall mortality was lower in the vitamin C supplemented group when compared to the control group, however, this difference was not statistically significant (RR 0.51; 95% CI 0.24 to 1.09; p = 0.052; *I*^2^ = *0*; p = 0.65) (Fig. 3). The Funnel plot (Fig. 4) and the regression-based Egger’s test for small-study effects, did not reveal apparent publication bias (p = 0.206). To assess the robustness of the findings and potential publication bias, we conducted a Fail-Safe calculation using the Rosenthal approach. This analysis suggested that an additional 5 studies with a similar effect size (RR = 0.51) would be needed to confirm the absence of publication bias.

#### Trial sequential analysis

The TSA graphs are presented in Supplementary Figs. S1, S2 and S3. Although a relatively large sample size of 908 would be required for a treatment effect of 40%, there was a trend towards significant mortality reduction in the vitamin C supplemented group when compared to control group (RR 0.57; 95% CI 0.28 to 1.17, p = 0.127, *I*^2^ = 0, p = 0.655). Sensitivity analysis using the Biggerstaff-Tweedie random effects model confirmed these findings (RR 0.57; 95% CI 0.44 to 0.74, p = 0.157, *I*^2^ = 0, p = 0.655). Similar trends were also observed for treatment effects of 30% and 20%, although much larger sample sizes, 1699 and 4012, respectively, would be needed to demonstrate a mortality reduction with vitamin C supplementation of patients being treated for pneumonia (Supplementary Figs. S2 and S3).

#### Subgroup analysis

##### Exclusion of COVID19 studies

After exclusion of COVID-19 studies^23,25^, the overall mortality remained lower in patients who were in the vitamin C supplemented group compared to the control group, but the difference remained statistically non-significant (RR 0.46; 95% CI 0.13 to 1.62; p = 0.131 *I*^2^ = 0; p = 0.593) (Supplementary Fig. S4). The funnel plot (Supplementary Fig. S5) and the Egger’s test did not reveal any apparent publication bias (p = 0.339). The fail-safe calculation using the Rosenthal approach suggested that additional 3 studies, each with a similar effect size (RR = 0.46) would be needed to confirm absence of publication bias.

#### Route of vitamin C administration

After exclusion of a study which used oral vitamin C^7^, the mortality remained lower among patients in the vitamin C supplemented group compared to those in the control group but was not statistically significant (RR 0.57; 95% CI 0.24 to 1.36; p = 0.122; *I*^2^ = 0, p = 0.64) Supplementary Fig. S6). The funnel plot (Supplementary Fig. S7) and the Egger’s test did not reveal any publication bias (p = 0.421). The fail-safe calculation using the Rosenthal approach suggested that 1 additional study with a similar effect size (RR = 0.57) would be needed to confirm absence of publication bias.

### Secondary outcomes

#### Hospital LOS

Only three of the six studies^10,23,25^ involving 179 patients determined the efficacy of vitamin C supplementation on hospital LOS. Patients who were in the vitamin C supplemented group had a shorter hospital LOS but this difference was not statistically significant and there was marked heterogeneity between studies (SMD – 0.23; 95% CI – 1.68 to 1.21; p = 0.558; *I*^2^ = 81.1%, p = 0.005) (Supplementary Fig. S8). The funnel plot (Supplementary Fig. S9) did show some evidence of publication bias, however, the Egger’s test was not significant (p = 0.810).

Exploration of heterogeneity by use of the leave-one-out sensitivity analysis identified one outlier^23^, an open-label randomized controlled trial (RCT). Upon exclusion of this study from the meta-analysis, a statistically significant reduction in hospital LOS was observed in patients receiving vitamin C supplementation compared to the control group, accompanied by a decrease in heterogeneity (SMD – 0.59; 95% CI – 0.96 to – 0.22; p = 0.001 *I*^2^ = 0, p = 0.36) (Supplementary Fig. S10). The funnel plot (Supplementary Fig. S11) revealed evidence of publication bias and the Egger’s test remained non-significant. To assess the robustness of findings regarding publication bias, a fail-safe calculation using the Rosenthal approach was performed. This calculation suggested that an additional 5 studies, each with a similar effect size (SMD = – 0.23), would be needed to confirm the absence of publication bias.

#### ICU LOS

Analysis of ICU LOS included data from only two of the six studies (Jamali Moghadam Siahkali et al.^23^; Mahmoodpoor et al.^22^), comprising a total of 140 patients. The comparison between patients in the vitamin C supplemented group and the control group did not reveal a statistically significant difference in ICU LOS, and the studies exhibited heterogeneity (standardized mean difference [SMD] – 0.13; 95% Confidence Interval [CI] – 3.77 to 3.52; p = 0.737; I^2^ = 64.2%, p = 0.09) (see Supplementary Fig. S12).

Given the limited number of studies, we refrained from exploring the sources of heterogeneity. Although the funnel plot (Supplementary Fig. S13) may not provide statistically informative insights due to the small sample size, it did not exhibit any apparent asymmetry.

#### Mechanical ventilation

Two studies^22,23^ involving 140 CAP patients determined the risk of intubation. There was no significant difference in intubation rates in the vitamin C supplemented group when compared to the control group (RR 0.77; 95% CI 0.00 to 122.06, p = 0.634, *I*^2^ = 2%, p = 0.312) (Supplementary Fig. S14). The funnel plot (Supplementary Fig. S15) did not show any publication bias and the Egger’s test could not be performed due to insufficient number of studies. The fail-safe calculation using the Rosenthal approach suggested that no additional study with a similar effect size (RR = 0.77) would be needed to confirm absence of publication bias.

#### Vasopressor use

Vasopressor use was reported by only one study^22^ which found that the duration of vasopressor use was significantly reduced among patients being treated for CAP who were in the vitamin C supplemented group when compared to placebo (2.28 ± 1.24 days vs. 3.39 ± 1.23 days, p = 0.003). There was no significant difference in the dose of vasopressor used between the two groups (6.8 ± 3.18 mcg/min vs. 8.26 ± 3.58 mcg/min, p = 0.14) in vitamin C and control groups, respectively.

#### Readmission risk

Only one study^10^ reported risk of 30-day readmissions which was not significantly different among treated CAP patients who received vitamin C compared to the control group (3% vs. 11% respectively, p = 0.22).

#### Time to clinical stabilisation

Time to clinical stability was reported by only one study^10^ which found a trend towards early stability among patients with CAP with the administration of vitamin C compared to the control group (median 22 h (IQR 40, 90) vs. 49 h (IQR 18, 137), p = 0.083).

#### Adverse effects of vitamin C

Only two studies^10,22^ reported data on adverse effects of supplementation of vitamin C. Chambers et al.^10^ reported 2 adverse events which could be possibly related to vitamin C administration including nausea and vomiting, while Mahmoodpoor et al.^22^ reported 3 episodes of hypotension and tachycardia during IV administration of vitamin C which were self-limited and resolved after reduction in dose of vitamin C. No study attributed AKI to administration of vitamin C. One study^10^ also reported minor adverse effects associated with the use of placebo, including nausea, vomiting and distaste for the medication.

## Discussion

This systematic review and meta-analyses evaluated the efficacy and safety of vitamin C in the treatment of patients with CAP. By including recent RCTs with a substantial number of CAP patients, our primary outcome analysis indicated a noteworthy trend towards a reduction in overall mortality in the vitamin C treatment group when compared to the control group. However, this observed reduction in mortality did not reach statistical significance, warranting careful interpretation of the findings. The TSA confirmed the trend towards reduced mortality in the vitamin C supplemented group but suggested that a sample size of 908 would be required to achieve statistical significance for a 40% reduction in relative risk.

After excluding studies^23,25^ which included only COVID-19 patients and an older study incorporating oral vitamin C supplementation, the non-significant reduction in mortality persisted between the two groups. The absence of statistical significance in our primary outcome may be attributed to the limited number of studies included in the meta-analysis. This highlights the critical need for future well-designed and adequately powered trials to offer more robust evidence regarding the role of vitamin C in CAP treatment. It is notable that a previous meta-analysis^28^ did not identify any studies investigating the mortality benefits of vitamin C in CAP.

Our systematic review contradicts a recent harmonised study^27^ combining data from two RCTs^26,29^ on vitamin C use in COVID-19 patients. This study suggests futility with vitamin C supplementation, but acknowledges limitations, including study design variations, post-randomisation care discrepancies, and lack of COVID-19 vaccination data. These limitations stress the need for cautious interpretation and highlight the importance of additional research to understand the relationship between vitamin C supplementation and outcomes in COVID-19 patients.

Our findings, although non-significant, align with a recent systematic review^30^ which included 24 RCTs and suggested that intravenous vitamin C might improve short-term mortality (RR 0.82; 95% CI 0.65 to 1.02; p = 0.07) and overall mortality (RR 0.86; 95% CI 0.74 to 1.01; p = 0.06) in septic patients; their review was not just limited to those with CAP. Despite these promising trends, it is essential to consider the limitations of the studies included in the above meta-analysis, such as high heterogeneity and publication bias. Thus, current evidence on the mortality benefits of vitamin C supplementation during treatment for severe infections, including CAP, remains inconclusive.

Our meta-analysis indicates a potential trend towards a reduction in hospital LOS in the vitamin C supplemented group compared to the control group. However, the studies included in our review exhibited significant heterogeneity. Following sensitivity analysis and the exclusion of an outlier study, our review revealed a statistically significant decrease in hospital LOS in the vitamin C supplemented group compared to the control group. It is crucial to approach this finding with caution due to the inclusion of a limited number of studies with smaller sample sizes in this review, raising the potential for inflated effect sizes as has been highlighted by Zhang et al.^31^.

In contrast, our analysis of ICU length of stay did not reveal a statistically significant difference between the vitamin C and control groups. While this non-significant finding could be influenced by the limited number of studies available for analysis and the observed heterogeneity in study designs, it remains possible that the beneficial effects of vitamin C supplementation are more visible in those less critically unwell. These results are, however, similar to a recent systematic review in septic patients which found that vitamin C did not reduce ICU LOS when compared to the control group (RR – 0.05; 95% CI – 0.19 to 0.09; p = 0.50).

This study suggests that the duration of vasopressor use was significantly shorter among critically ill CAP patients who received vitamin C. However, only one study was available for review, preventing a meta-analysis. Nonetheless, a prior meta-analysis by Muhammad et al.^32^, that included twelve studies on septic patients also reported a statistically significant reduction in vasopressor support time for those treated with vitamin C compared to placebo (SMD = – 1.03; 95% CI – 1.62 to – 0.44; p = 0.001; I^2^ = 88.96%). The substantial heterogeneity observed in the studies included in this meta-analysis limits the certainty of the evidence. Therefore, further research is needed to explore the role of vitamin C in reducing vasopressor support among septic patients.

A critical consideration in our analysis is the overall poor quality of the included studies, with the exception of two studies^10,22^. The methodological limitations and potential biases in the majority of studies underscore the need for rigorously conducted trials to enhance the reliability of our findings.

Recent research^26,32^ has primarily focused on the efficacy of vitamin C in critically ill septic patients, who are inherently at a heightened risk for adverse clinical outcomes. There has also been an ongoing debate about the choice of vitamin C preparation used in these studies, with concerns raised that using ascorbic acid instead of sodium ascorbate may exacerbate metabolic acidosis and lead to poorer outcomes^33^. Furthermore, the abrupt discontinuation of vitamin C supplementation could potentially cause a further decline in plasma vitamin C levels, resulting in a rebound increase in oxidant stress^34^. This has contributed to lingering clinical equipoise regarding the benefits of vitamin C in patients with sepsis.

Urgent future research is needed to elucidate the differential effects of vitamin C supplementation in pneumonia and sepsis, considering factors such as dosage, route of administration (intravenous vs. oral), and type of preparation used (sodium ascorbate vs. ascorbic acid). Additionally, exploring a slow tapering regimen instead of abrupt discontinuation could help maintain stable serum vitamin C levels. In addition, more studies are needed to ascertain benefits of vitamin C among non-critically ill patients to determine whether it can prevent further clinical deterioration.

Moreover, future studies should incorporate the vaccination status of participants to better understand the impact of vitamin C on patient outcomes. Considering the potentially protective role of pneumococcal vaccination in patients with CAP^35^, this could be a crucial variable to consider in future research.

## Conclusions

In conclusion, our study offers insights into potential benefits of vitamin C supplementation in CAP treatment. While an apparent reduction in overall mortality and hospital LOS was observed, the lack of statistical significance and poor study quality, necessitates cautious interpretation. Our findings emphasise the need for rigorous trials to clarify vitamin C’s true impact on CAP outcomes.

### Supplementary Information

Supplementary Information 1.

Supplementary Information 2.

Supplementary Information 3.

## Data Availability

Data used for this study are available from the corresponding author on request.
