# Supplementary material for: Efficacy and safety of vitamin C supplementation in the treatment of community-acquired pneumonia: a systematic review and meta-analysis with trial sequential analysis
Source: Sci Rep. 2024 May 24;14:11846. doi: 10.1038/s41598-024-62571-5 (PMC11116443; doi:10.1038/s41598-024-62571-5)
Supplement: Supplementary file 1 — Supplementary Information 1. [file 41598_2024_62571_MOESM1_ESM.zip › Supplementary Fig(Int J Inf Ds)/Figure S8.pdf]

Study	Experimental Mean	SD	Total	Control Mean	SD	Total	Weight	Std. Mean Difference IV, Random, 95% CI
Chambers et al 2023	2.90	2.1000	36	5.00	3.4000	39	34.9%	−0.73 [−1.20; −0.26]
JamaliMoghadamSiahkali et al 2021	8.50	3.7000	30	6.50	5.9200	30	33.8%	0.40 [−0.11; 0.91]
Tehrani et al 2022	14.00	8.0000	18	17.00	8.0000	26	31.3%	−0.37 [−0.97; 0.24]
**Total (95% CI)**			**84**			**95**	**100.0%**	**−0.23 [−1.68; 1.21]**

Heterogeneity: $Tau^2 = 0.2762$; $Chi^2 = 10.36$, df = 2 ($P < 0.01$); $I^2 = 81\%$

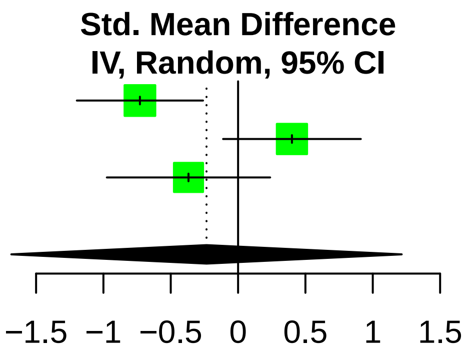

Supplementary Figure S8 Forest plot comparing hospital length of stay between vitamin C supplemented group and control group
