# Supplementary material for: Efficacy and safety of vitamin C supplementation in the treatment of community-acquired pneumonia: a systematic review and meta-analysis with trial sequential analysis
Source: Sci Rep. 2024 May 24;14:11846. doi: 10.1038/s41598-024-62571-5 (PMC11116443; doi:10.1038/s41598-024-62571-5)
Supplement: Supplementary file 1 — Supplementary Information 1. [file 41598_2024_62571_MOESM1_ESM.zip › Supplementary Fig(Int J Inf Ds)/Figure S14.pdf]

Study	Experimental Events	Experimental Total	Control Events	Control Total	Weight	Risk Ratio IV, Random, 95% CI
JamaliMoghadamSiahkali 2021	5	30	4	30	40.7%	1.25 [0.37; 4.21]
Mahmoodpoor et al 2021	5	40	9	40	59.3%	0.56 [0.20; 1.51]
**Total (95% CI)**		**70**		**70**	**100.0%**	**0.77 [0.00; 122.06]**

Heterogeneity: $Tau^2 = 0.0066$; $Chi^2 = 1.02$, df = 1 (P = 0.31); $I^2 = 2\%$

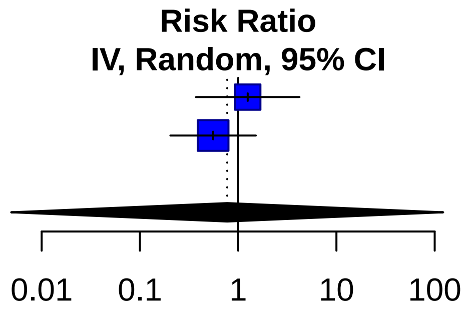

Supplementary Figure S14 Forest plot comparing risk of intubation between vitamin C supplemented group and control group
