# Supplementary material for: Efficacy and safety of vitamin C supplementation in the treatment of community-acquired pneumonia: a systematic review and meta-analysis with trial sequential analysis
Source: Sci Rep. 2024 May 24;14:11846. doi: 10.1038/s41598-024-62571-5 (PMC11116443; doi:10.1038/s41598-024-62571-5)
Supplement: Supplementary file 1 — Supplementary Information 1. [file 41598_2024_62571_MOESM1_ESM.zip › Supplementary Fig(Int J Inf Ds)/Figure S12.pdf]

Study	Experimental Mean	SD	Total	Control Mean	SD	Total	Weight	Std. Mean Difference IV, Random, 95% CI
JamaliMoghadamSiahkali et al 2021	5.50	3.7000	30	5.00	1.4800	30	47.6%	0.18 [−0.33; 0.68]
Mahmoodpoor et al 2021	12.77	3.7100	40	14.15	3.1200	40	52.4%	−0.40 [−0.84; 0.04]
**Total (95% CI)**			**70**			**70**	**100.0%**	**−0.13 [−3.77; 3.52]**

Heterogeneity: $Tau^2 = 0.1057$; $Chi^2 = 2.79$, df = 1 (P = 0.09); $I^2 = 64\%$

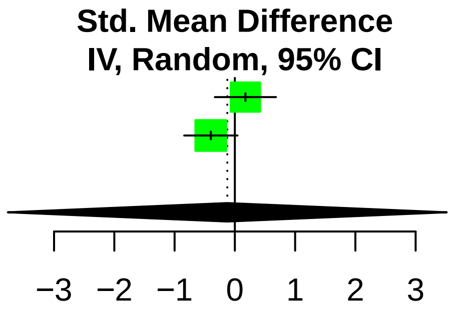

Supplementary Figure S12 Forest plot comparing intensive care unit (ICU) length of stay between vitamin C supplemented group and control group
