# Supplementary material for: Efficacy and safety of vitamin C supplementation in the treatment of community-acquired pneumonia: a systematic review and meta-analysis with trial sequential analysis
Source: Sci Rep. 2024 May 24;14:11846. doi: 10.1038/s41598-024-62571-5 (PMC11116443; doi:10.1038/s41598-024-62571-5)
Supplement: Supplementary file 1 — Supplementary Information 1. [file 41598_2024_62571_MOESM1_ESM.zip › Supplementary Fig(Int J Inf Ds)/Figure S2.pdf]

Relative Risk Reduction: 30%

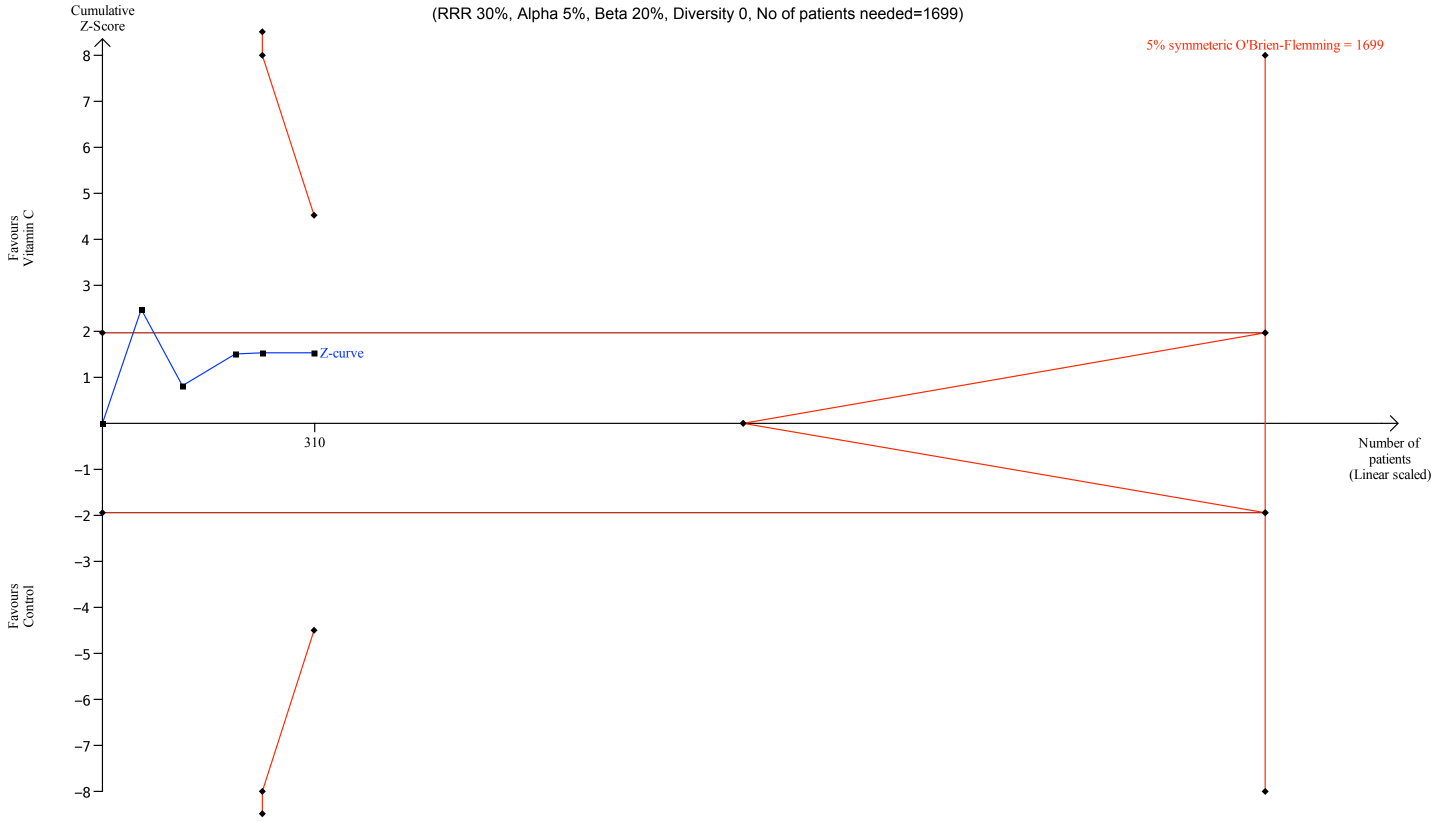

Figure S2 Trial Sequence Analysis graph showing number of patients required for relative reduction in mortality of 30%
