# Supplementary material for: Efficacy and safety of vitamin C supplementation in the treatment of community-acquired pneumonia: a systematic review and meta-analysis with trial sequential analysis
Source: Sci Rep. 2024 May 24;14:11846. doi: 10.1038/s41598-024-62571-5 (PMC11116443; doi:10.1038/s41598-024-62571-5)
Supplement: Supplementary file 1 — Supplementary Information 1. [file 41598_2024_62571_MOESM1_ESM.zip › Supplementary Fig(Int J Inf Ds)/Figure S6.pdf]

Study	Experimental Events	Experimental Total	Control Events	Control Total	Weight	Risk Ratio IV, Random, 95% CI
Chambers et al 2023	0	36	2	39	5.7%	0.22 [0.01; 4.36]
JamaliMoghadamSiahkali et al 2021	3	30	3	30	22.5%	1.00 [0.22; 4.56]
Mahmoodpoor et al 2021	6	38	11	40	65.4%	0.57 [0.24; 1.40]
Tehrani et al 2022	0	18	4	26	6.3%	0.16 [0.01; 2.78]
**Total (95% CI)**		**122**		**135**	**100.0%**	**0.57 [0.24; 1.36]**

Heterogeneity: $Tau^2 = 0$; $Chi^2 = 1.69$, df = 3 (P = 0.64); $I^2 = 0\%$

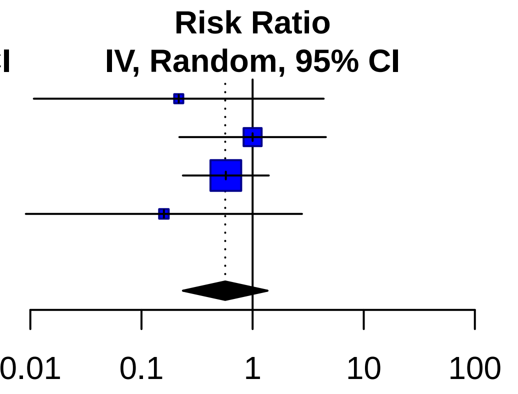

Supplementary Figure S6 Forest plot showing comparison of overall mortality between vitamin C supplemented group and control group after exclusion of studies using oral vitamin C. CI; confidence interval
