# Supplementary figures and images for: Efficacy and safety of vitamin C supplementation in the treatment of community-acquired pneumonia: a systematic review and meta-analysis with trial sequential analysis
Source: Sci Rep. 2024 May 24;14:11846. doi: 10.1038/s41598-024-62571-5 (PMC11116443; doi:10.1038/s41598-024-62571-5)

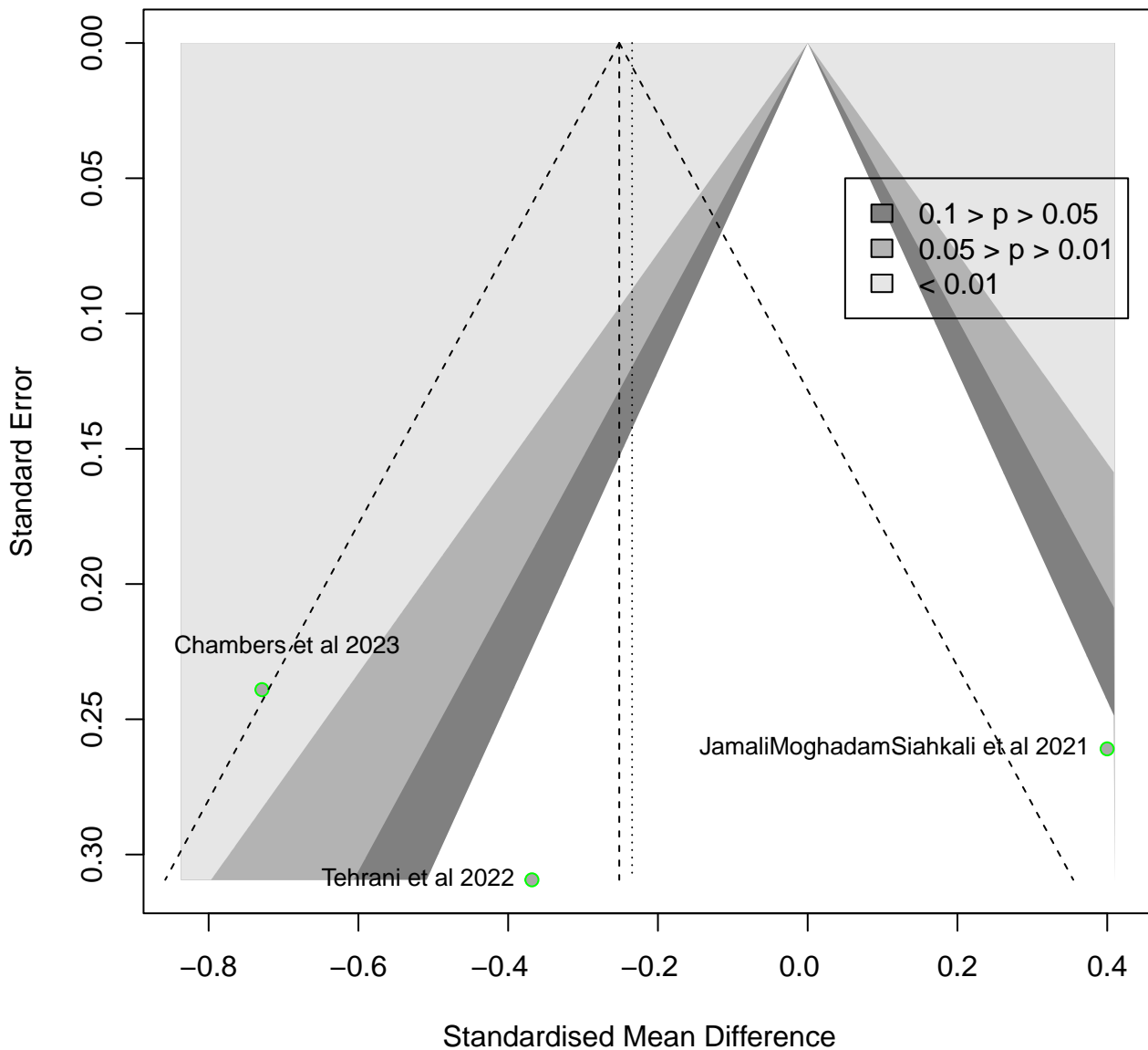

Supplement: Supplementary file 1 — Supplementary Information 1. [file 41598_2024_62571_MOESM1_ESM.zip › Supplementary Fig(Int J Inf Ds)/Figure S9.pdf]

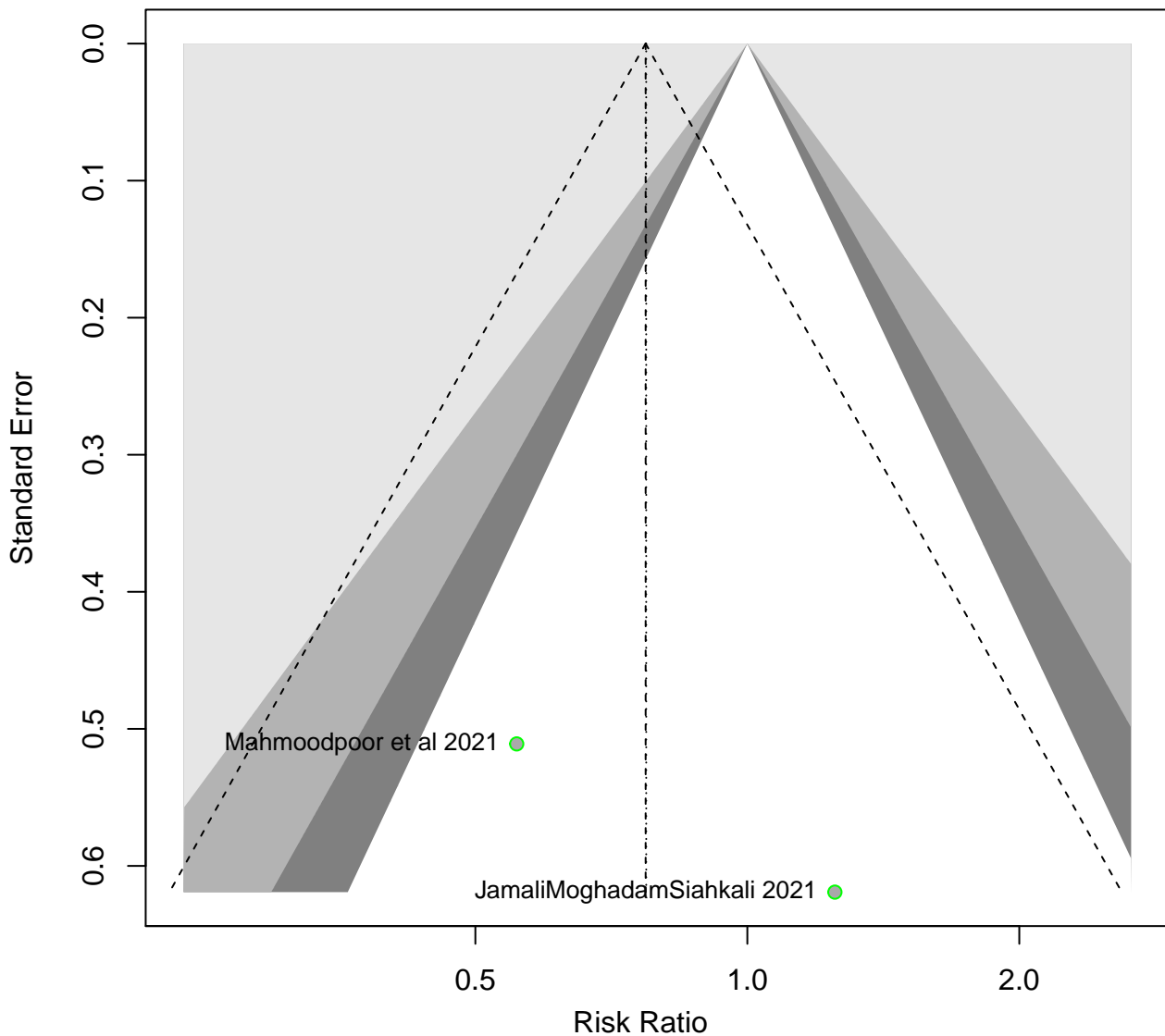

Supplement: Supplementary file 1 — Supplementary Information 1. [file 41598_2024_62571_MOESM1_ESM.zip › Supplementary Fig(Int J Inf Ds)/Figure S15.pdf]

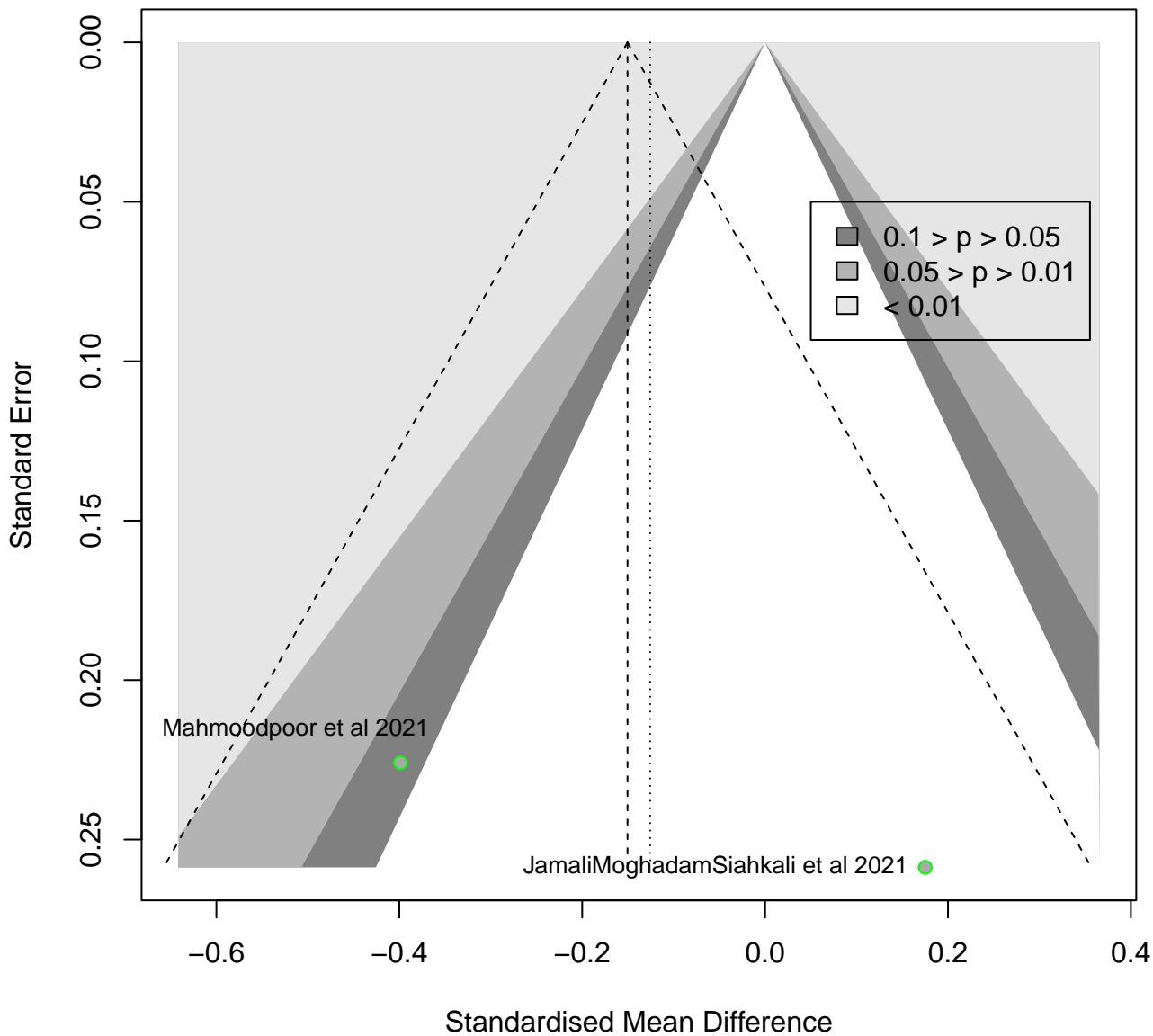

**Supplementary Figure S13** Funnel plot for intensive care unit (ICU) length of stay

Supplement: Supplementary file 1 — Supplementary Information 1. [file 41598_2024_62571_MOESM1_ESM.zip › Supplementary Fig(Int J Inf Ds)/Figure S13.pdf]

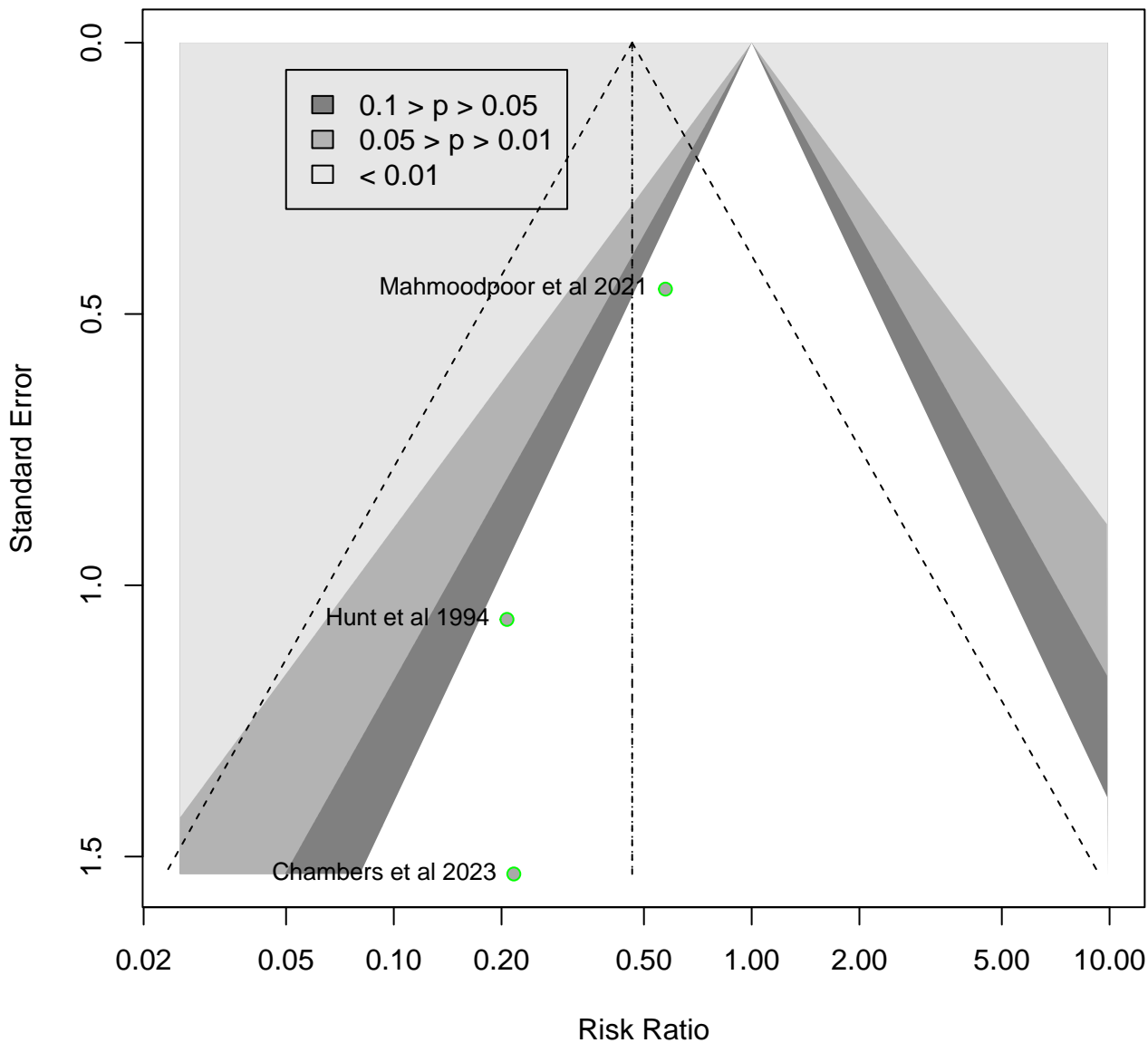

**Figure S5** Funnel plot for overall mortality excluding COVID-19 studies

Supplement: Supplementary file 1 — Supplementary Information 1. [file 41598_2024_62571_MOESM1_ESM.zip › Supplementary Fig(Int J Inf Ds)/Figure S5.pdf]
