# Supplementary material for: Efficacy and safety of vitamin C supplementation in the treatment of community-acquired pneumonia: a systematic review and meta-analysis with trial sequential analysis
Source: Sci Rep. 2024 May 24;14:11846. doi: 10.1038/s41598-024-62571-5 (PMC11116443; doi:10.1038/s41598-024-62571-5)
Supplement: Supplementary file 2 — Supplementary Information 2. [file 41598_2024_62571_MOESM2_ESM.docx]

**Search strategy for the study “*Efficacy and Safety of Vitamin C Supplementation in the Treatment of Community-Acquired Pneumonia: A Systematic Review and Meta-Analysis with Trial Sequential Analysis”***

## **Database(s): Ovid MEDLINE(R)**

Search Strategy:

| # | Searches |
| --- | --- |
| 1 | adult/ or aged/ or "aged, 80 and over"/ or frail elderly/ or middle aged/ or young adult/ |
| 2 | (adult* or (aged adj3 (">18" or "24-44" or "60" or "65" or "over 80*" or "18-60 years"))  or "middle aged" or elderly or "older adult*" or "older person*").tw,kf. |
| 3 | 1 or 2 |
| 4 | pneumonia/ or bronchopneumonia/ or pneumonia, bacterial/ or pneumonia, viral/ |
| 5 | (pneumonia or bronchopneumonia or "lower respiratory tract infection*").tw,kf. |
| 6 | 4 or 5 |
| 7 | Hospitalization/ |
| 8 | Inpatients/ |
| 9 | Critical Care/ |
| 10 | (Hospiti?ation or inpatient* or patient* or ICCU or "Intensive care unit" or "critical  care").tw,kf. |
| 11 | or/7-10 |
| 12 | Ascorbic Acid/ |
| 13 | ("vitamin C" or "Ascorbic Acid" or ascorbate).tw,kf. |
| 14 | 12 or 13 |
| 15 | 3 and 6 and 11 and 14 |
| 16 | Randomized Controlled Trials as Topic/ or Randomized Controlled Trial/ |
| 17 | random*.pt. or random*.mp. |
| 18 | (placebo or groups or RCT* or "Double blind").tw,kf. |

| 19 | or/16-18 |
| --- | --- |
| 20 | 15 and 19 |

**Database CINAHL**

| # | Query |
| --- | --- |
| S20 | S15 AND S19 |
| S19 | S16 OR S17 OR S18 |
| S18 | TI ( (placebo or groups or RCT* or "Double blind") ) OR AB ( (placebo or groups or RCT* or "Double blind") ) |
| S17 | PT random* OR random* |
| S16 | (MH "Randomized Controlled Trials") |
| S15 | S3 AND S6 AND S11 AND S14 |
| S14 | S12 OR S13 |
| S13 | TI ( ("vitamin C" or "Ascorbic Acid" or ascorbate) ) OR AB ( ("vitamin C" or "Ascorbic Acid" or ascorbate) ) |
| S12 | (MH "Ascorbic Acid") |
| S11 | S7 OR S8 OR S9 OR S10 |
| S10 | TI ( (Hospiti?ation or inpatient* or patient* or ICCU or "Intensive care unit" or "critical care") ) OR AB ( (Hospiti?ation or inpatient* or patient* or ICCU or "Intensive care unit" or "critical care") ) |
| S9 | (MH "Critical Care") |
| S8 | (MH "Inpatients") |
| S7 | (MH "Hospitalization") |
| S6 | S4 OR S5 |
| S5 | TI ( (pneumonia or bronchopneumonia or "lower respiratory tract infection*") ) OR AB ( (pneumonia or bronchopneumonia or "lower respiratory tract infection*") ) |
| S4 | (MH "Pneumonia") OR (MH "Bronchopneumonia") OR (MH "Pneumonia, Bacterial") OR (MH "Pneumonia, Viral") |
| S3 | S1 OR S2 |
| S2 | TI ( (adult* or (aged N2 (">18" or "24-44" or "60" or "65" or "over 80*" or "18- 60 years")) or "middle aged" or elderly or "older adult*" or "older person*") ) OR AB ( (adult* or (aged N2 (">18" or "24-44" or "60" or "65" or "over 80*" or "18- 60 years")) or "middle aged" or elderly or "older adult*" or "older person*") ) |

# 2

| S1 | (MH "Aged") OR (MH "Adult") OR (MH "Aged, 80 and Over") OR (MH "Frail Elderly") OR (MH "Middle Age") |
| --- | --- |

## **Database: Scopus**

( TITLE-ABS-KEY ( ( adult OR ( aged W/2 ( ">18" OR "24-44" OR "60" OR "65" OR "over 80" OR

"18-60 years" ) ) OR "middle aged" OR elderly OR "older adult" OR "older person" ) ) AND TITLE- ABS-KEY ( ( pneumonia OR bronchopneumonia OR "lower respiratory tract infection" ) ) AND TITLE-ABS-KEY ( ( hospitalization OR inpatient OR patient OR iccu OR "Intensive care unit" OR "critical care" ) ) AND TITLE-ABS-KEY ( ( "vitamin C" OR "Ascorbic Acid" OR ascorbate ) ) AND TITLE-ABS-KEY ( ( random* OR rct OR placebo OR "double blind" OR group ) ) )

##
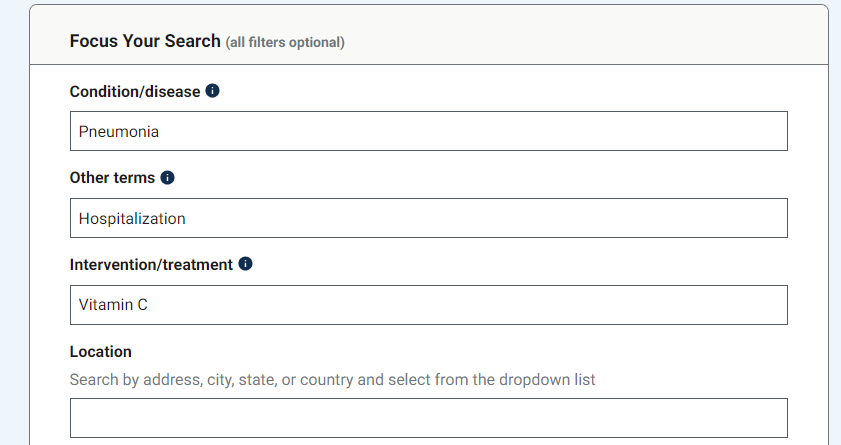
**Database: ClinicalTrials.gov**

**Cochrane library Search box 1**

(adult* or (aged NEAR/2 (">18" or "24-44" or "60" or "65" or "over 80" or "18-60 years")) or "middle aged" or elderly or "older adult" or "older adults" OR "older persons" OR "older person*")

## Search box 2

(pneumonia or bronchopneumonia or "lower respiratory tract infection*")

**Search box 3**

# 3

(Hospiti?ation or inpatient* or patient* or ICCU or "Intensive care unit" or "critical care")

## Search box 4

("vitamin C" or "Ascorbic Acid")

## Search box 5

(Random* OR RCT* OR placebo OR "double blind" OR group*
