# Supplementary material for: Efficacy and safety of vitamin C supplementation in the treatment of community-acquired pneumonia: a systematic review and meta-analysis with trial sequential analysis
Source: Sci Rep. 2024 May 24;14:11846. doi: 10.1038/s41598-024-62571-5 (PMC11116443; doi:10.1038/s41598-024-62571-5)
Supplement: Supplementary file 3 — Supplementary Information 3. [file 41598_2024_62571_MOESM3_ESM.docx]

**Supplement 3** Table showing excluded studies and reasons for exclusion

| **Study ID** | **Reasons for exclusion** | **Details** |
| --- | --- | --- |
| [26] | Only included patients with septic shock | The source of sepsis in two-thirds of patients was extra-pulmonary making it inconsistent with our inclusion criteria |
| [27] | Harmonised trial focused on COVID-19 patients and amalgated data from study [26] and another trial [29] | Used a range of unblinded interventions to treat COVID-19 patients, rendering it beyond the scope of this systematic review |

COVID-19, corona virus disease-2019
